# Supplementary material for: Dissociation between phase and power correlation networks in the human brain is driven by co-occurrent bursts
Source: Commun Biol. 2023 Mar 18;6:286. doi: 10.1038/s42003-023-04648-x (PMC10024695; doi:10.1038/s42003-023-04648-x)
Supplement: Supplementary file 2 — Supplementary Material [file 42003_2023_4648_MOESM2_ESM.pdf]

Supplementary items: Dissociation between phase and power correlation networks in the human brain is driven by co-occurrent bursts

February 9, 2023

Supplementary Note 1: Spontaneous cortical oscillations are super-Gaussian

A prominent feature of spontaneous cortical activity during wakefulness is the presence of alpha and beta rhythms. These can be observed in the spectral domain as peaks at ≈ 10 Hz and ≈ 16 Hz, respectively (see Supplementary Figure 1a). To analyse spatial patterns of excess cokurtosis in empirical data, we first quantify spatial patterns of large fluctuations in terms of excess kurtosis in empirical MEG data. We computed the excess kurtosis of source-reconstructed cortical signals at the alpha (≈ 10 Hz) and beta (≈ 16 Hz) peak-frequencies at all cortical regions-of-interest and averaged the obtained values over the 89 subjects.

Supplementary Figures 1b and c show the subject-averaged map of excess kurtosis in the alpha and beta frequency band, respectively. In the alpha band, the largest values are observed in the inferior parietal cortical and throughout posterior regions, including visual and auditory cortices and the precuneus, as well as in the somatosensory and primary motor cortices. In contrast, no high values are observed in frontal cortices and there appears to be a strict border formed by the central sulcus. In the beta band, the largest values were observed in the inferior parietal and lateral prefrontal cortices, and in the motor and somatosensory cortices (see Supplementary Figure 1c). The parietal and prefrontal regions constitute the well-known fronto-parietal attention network. In previous studies, this network was extracted from MEG data using either independent component or amplitude envelope correlation analysis, both of which exploit statistical relations between signals from different cortical regions. Our results demonstrate that a higher-cognitive resting-state network, the frontal-partietal attention network, can be detected based on an intrinsic property of the individual signals.

Supplementary Figure 1d shows the distribution of the real part of the signal in the alpha band originating from the left primary somatosensory cortex, which had the highest excess kurtosis. The corresponding distribution obtained from a randomized copy of the MEG sensor data is only shown. The observed distribution has longer tails than expected from a Gaussian signal with the same mean and variance, i.e. it is super-Gaussian. As an illustration, Figure 1e shows a ten-second epoch of this signal, together with the randomized copy. Both signals were z-scored. The observed signal crosses the three-sigma threshold more frequently than the randomized signal does, which is a manifestation of the signals' super-Gaussian nature.

The observed kurtosis maps were highly reproducible in a separate recording session (see Suppementary Figure 2a and b). To ensure that the subject-averaged maps were not dominated by those of a small number of subjects, we recalculated them after excluding the 20 subjects whose individual maps had the largest Euclidean norms. The spatial correlation between these maps and the original

maps was 0.99 (alpha band) and 0.97 (beta band). We also did this by excluding the 20 subjects whose maps had the largest maximal value over all cortical regions, yielding correlation coefficients of 0.99 (alpha band) and 0.98 (beta band). To exclude the possibility that the maps are driven by higher signal-to-noise ratios in posterior regions or are an artifact of the processing steps applied to the MEG sensor signals, we also calculated the maps on randomized MEG sensor signals, which were constructed to have the same cross-spectral matrix as the original signals, but are Gaussian. The obtained maps did not exhibit a clear spatial pattern (see Supplementary Figure 2c and d), were not reproducible (see Supplementary Figure 2e and f) and, throughout the cortex, the kurtosis values were about an order of magnitude smaller than those of the observed maps.

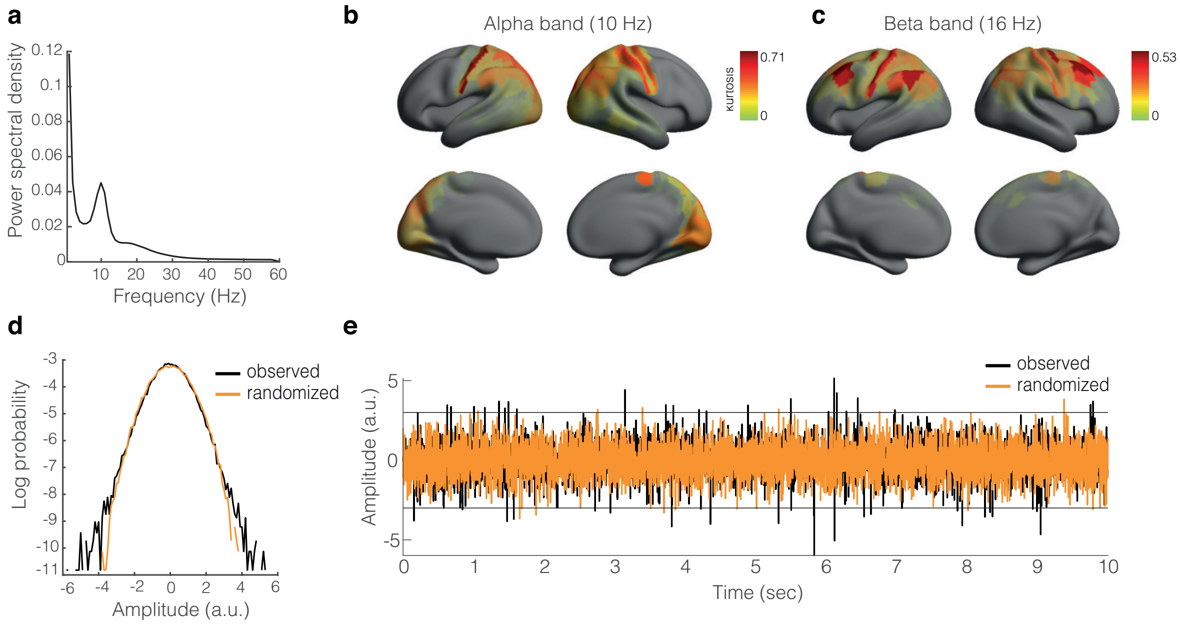

Supplementary Figure 1: Super-Gaussiannity of spontaneous cortical signals. (a) Power spectrum of the resting-state MEG sensor data, obtained by averaging spectra over MEG channels and subjects. **(b)** Excess kurtosis of the source-projected MEG signals at the alpha peak-frequency ($\approx$ 10 Hz). **(c)** Excess kurtosis of the source-projected MEG signals at the beta peak-frequency ($\approx$ 16 Hz). **(d)** Distribution of the real part of the Fourier coefficients in the alpha band of the signal originating from the left primary somatosensory cortex (black curve), together with a randomized copy (orange curve). The distribution was obtained by pooling the Fourier coefficients of all subjects. Before pooling, the coefficients were normalized to have unit variance. **(e)** Ten-second epoch of the observed (black trace) and randomized (orange trace) signal in the alpha band from the left primary somatosensory cortex. The signals are normalized to unit variance. The horizontal black lines denote

three standard-deviations. For better visibility, the cortical maps were thresholded at their average values.

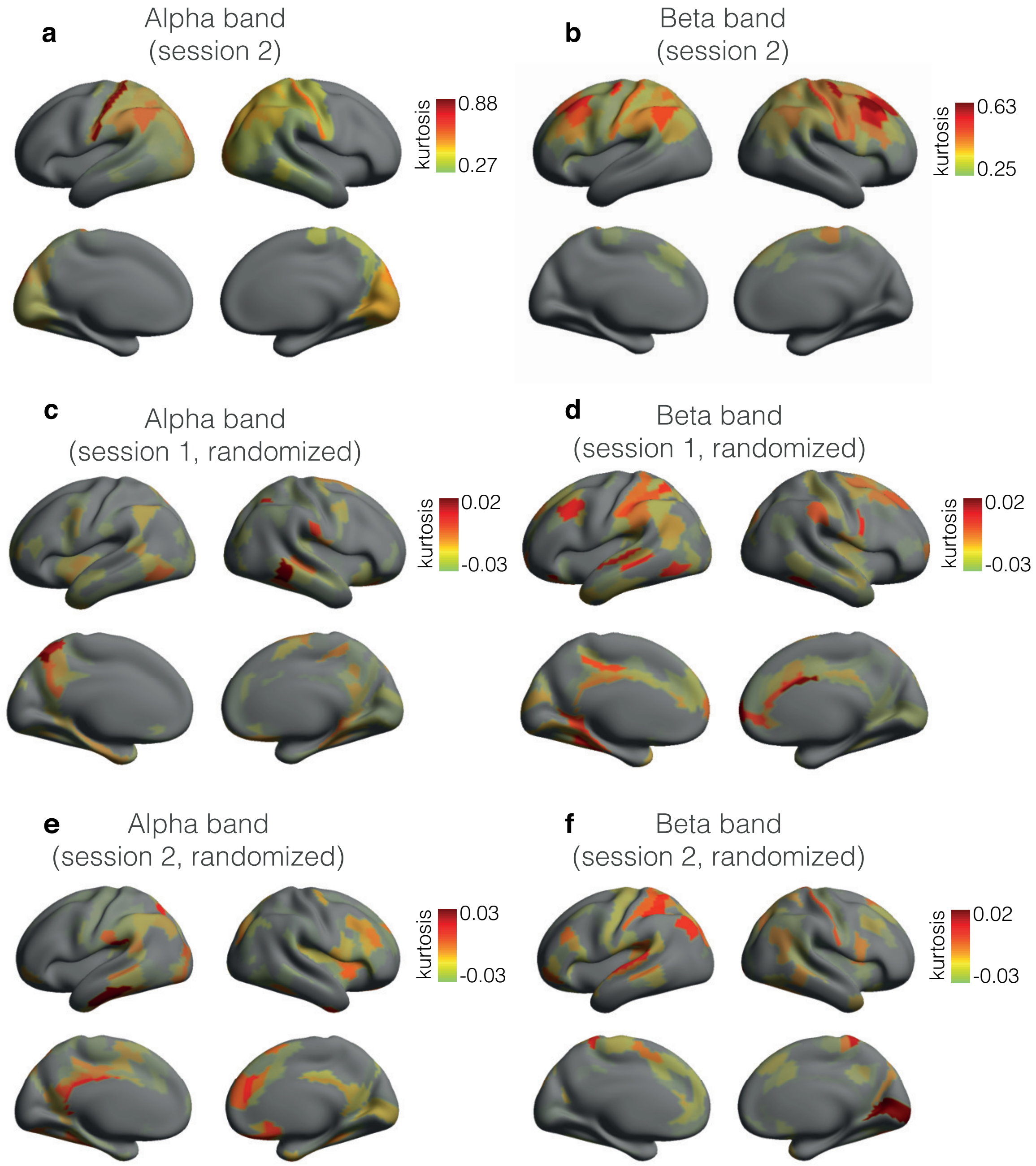

Supplementary Figure 2: Cortical kurtosis maps (a) Excess kurtosis in the alpha frequency band obtained from recording session 2. **(b)** Excess kurtosis in the beta frequency band obtained from recording session 2. **(c)** Excess kurtosis in the alpha frequency band obtained from randomized sensor signals of recording session 1. **(d)** Excess kurtosis in the beta frequency band obtained from randomized sensor signals of recording session 1. **(e)** Excess kurtosis in the alpha frequency band

obtained from randomized sensor signals of recording session 2. **(f)** Excess kurtosis in the beta frequency band obtained from randomized sensor signals of recording session 2. **Reproducibility:** The spatial correlation between the maps of the first and second recording sessions was 0.97 (alpha band) and 0.95 (beta band) which shows that they are highly reproducible. The spatial correlation between the randomized maps of the first and second recording sessions was 0.04 (alpha band) and 0.03 (beta band) which shows that they are not reproducible.

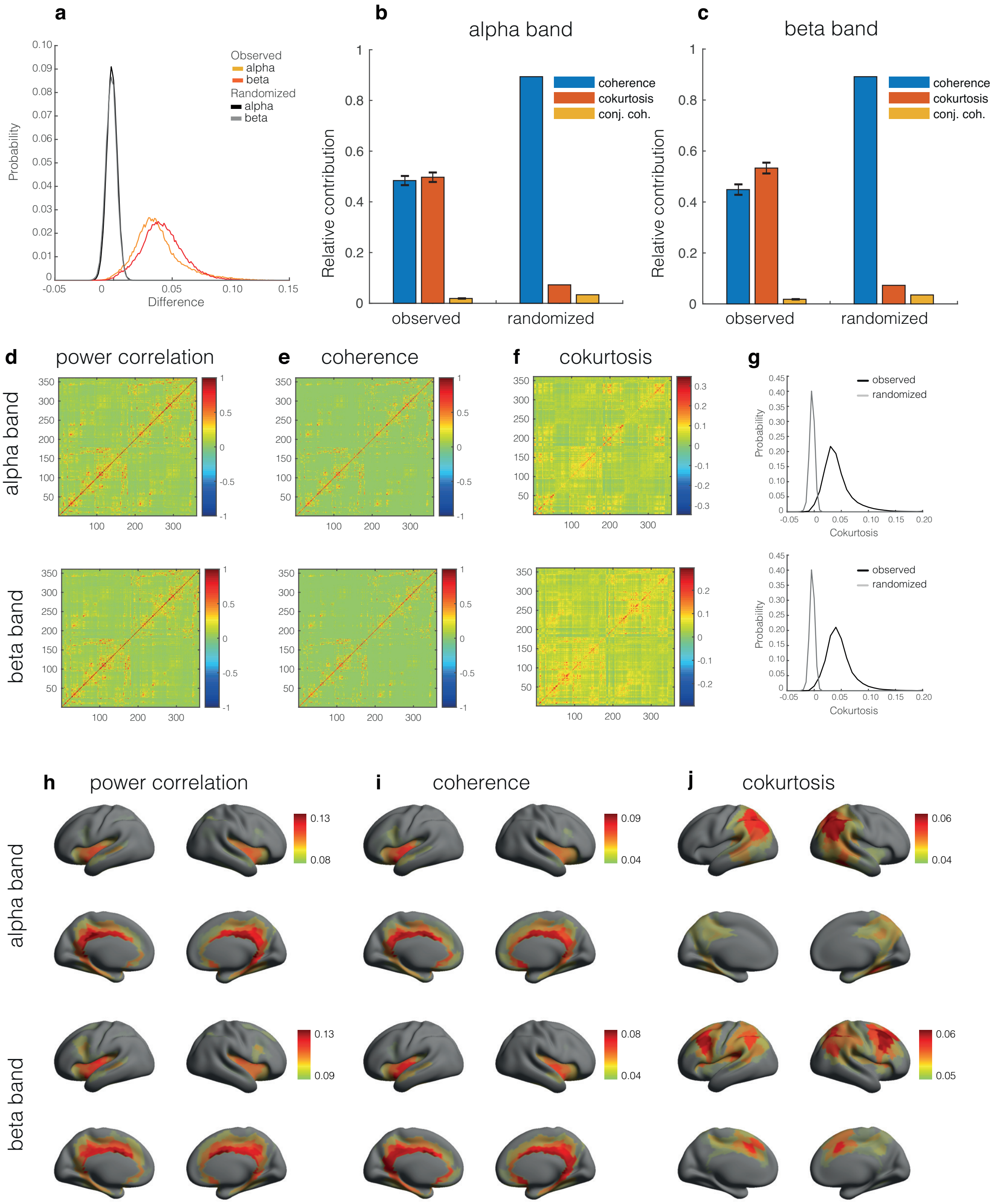

Supplementary Figure 3: Dissociation between cortical coherence and power correlation networks (second recording session) (a) Distributions of the observed differences (power correlation minus squared magnitude coherence) in the alpha (yellow curve) and beta (orange curve)

frequency bands and the corresponding null-distributions in black (alpha band) and grey (beta band). **(b)** Relative contributions of coherence (orange bars), cokurtosis (yellow bars), and conjugate coherence (black bars) to cortical power correlations in the alpha frequency band. **(c)** Same format as (b) but for the beta frequency band. The error bars of the observed contributions in panels **(b)** and **(c)** correspond to standard errors and were obtained by bootstrapping over subjects. Error bars for the randomized contributions are not displayed since their standard errors were negligible (less than 0.001). **(d)** Power correlation network in the alpha (top panel) and beta (bottom panel) frequency band. **(e)** Coherence network in the alpha(top panel) and beta (bottom panel) frequency band. **(f)** Cokurtosis network in the alpha (top panel) and beta (bottom panel)frequency band. The networks were obtained by averaging the respective subject-specific network matrices. **(g)** Distribution of the cokurtosis values in the alpha (top panel) and beta (bottom panel) frequency band (black curve) and of those of a randomized copy of the MEG signals (grey curve). **(h)** Color-coded cortical map of the region-averaged power correlation network matrix in the alpha (top panel) and beta (bottom panel) frequency band. **(i)** Color-coded cortical map of the region-averaged coherence network matrix in the alpha (top panel) and beta (bottom panel) frequency band. **(j)** Color-coded cortical map of the region-averaged cokurtosis network matrix in the alpha (top panel) and beta (bottom panel) frequency band. For better visibility, the cortical maps were thresholded at their average values. **Reproducibility:** The correlation between the vectorized upper triangular parts of the networks obtained from recording sessions 1 and 2 in the alpha band was 1.00 (power correlation), 1.00 (coherence), and 0.95 (cokurtosis). The correlation between the vectorized upper triangular parts of the networks obtained from recording sessions 1 and 2 in the beta band was 1.00 (power correlation), 1.00 (coherence), and 0.94 (cokurtosis). These results demonstrate that the statistical uncertainty in the estimated group-level network matrices is practically zero. The correlation between the seed-based cortical maps obtained from recording sessions 1 and 2 in the alpha band was 1.00 (power correlation), 1.00 (coherence), and 0.95 (cokurtosis). The correlation between the seed-based cortical maps obtained from recording sessions 1 and 2 in the beta band was 1.00 (power correlation), 1.00 (coherence), and 0.94 (cokurtosis). These results demonstrate that the statistical uncertainty in the estimated seed-based cortical maps is practically zero.

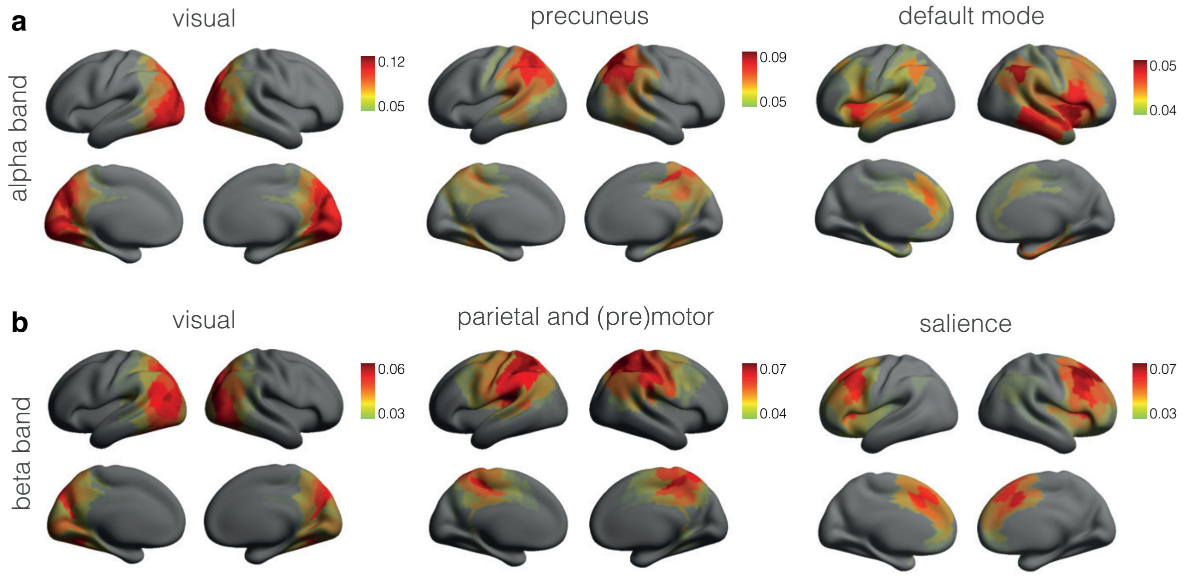

Supplementary Figure 4: Non-Gaussian power correlation networks (second recording session) (a) Non-Gaussian power correlation networks extracted from spontaneous cortical oscillations in the alpha frequency band. **(b)** Non-Gaussian power correlation networks extracted from spontaneous cortical oscillations in the beta frequency band. The networks in both panels were extracted by k-means clustering of the group-level seed-based cokurtosis matrices in the respective frequency bands. Displayed are the cluster centers. The number of clusters was determined by the elbow method applied to the average within-cluster sum of squared distances to the cluster centers. The ordering of the networks is the same as that in the first recording session (see Figure 4 of the main text).

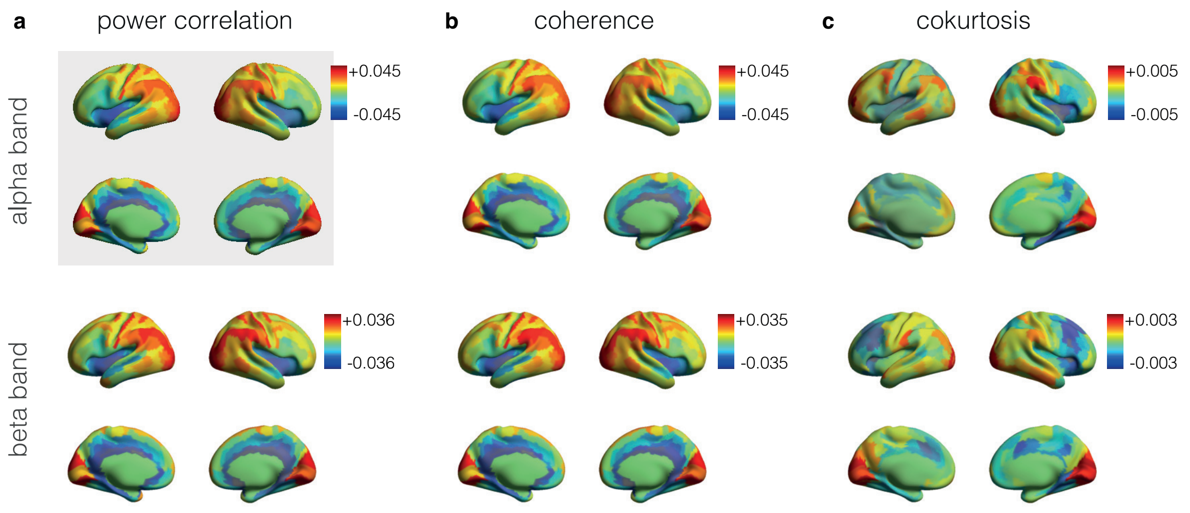

Supplementary Figure 5: Effect of signal orthogonalization (cortical difference maps) (a) Cortical difference map of the power correlation in the alpha (top) and beta (bottom) frequency band, i.e. the group-level power correlation map obtained from the orthogonalized signals minus the group-level power correlation map obtained from the non-orthogonalized signals. **(b)** Same format as **(a)** but for the coherence maps. **(c)** Same format as **(a)** but for the cokurtosis maps. Because the maps obtained from the orthogonalized and non-orthogonalized signals have different spatial averages, the spatial averages were subtracted before taking differences.

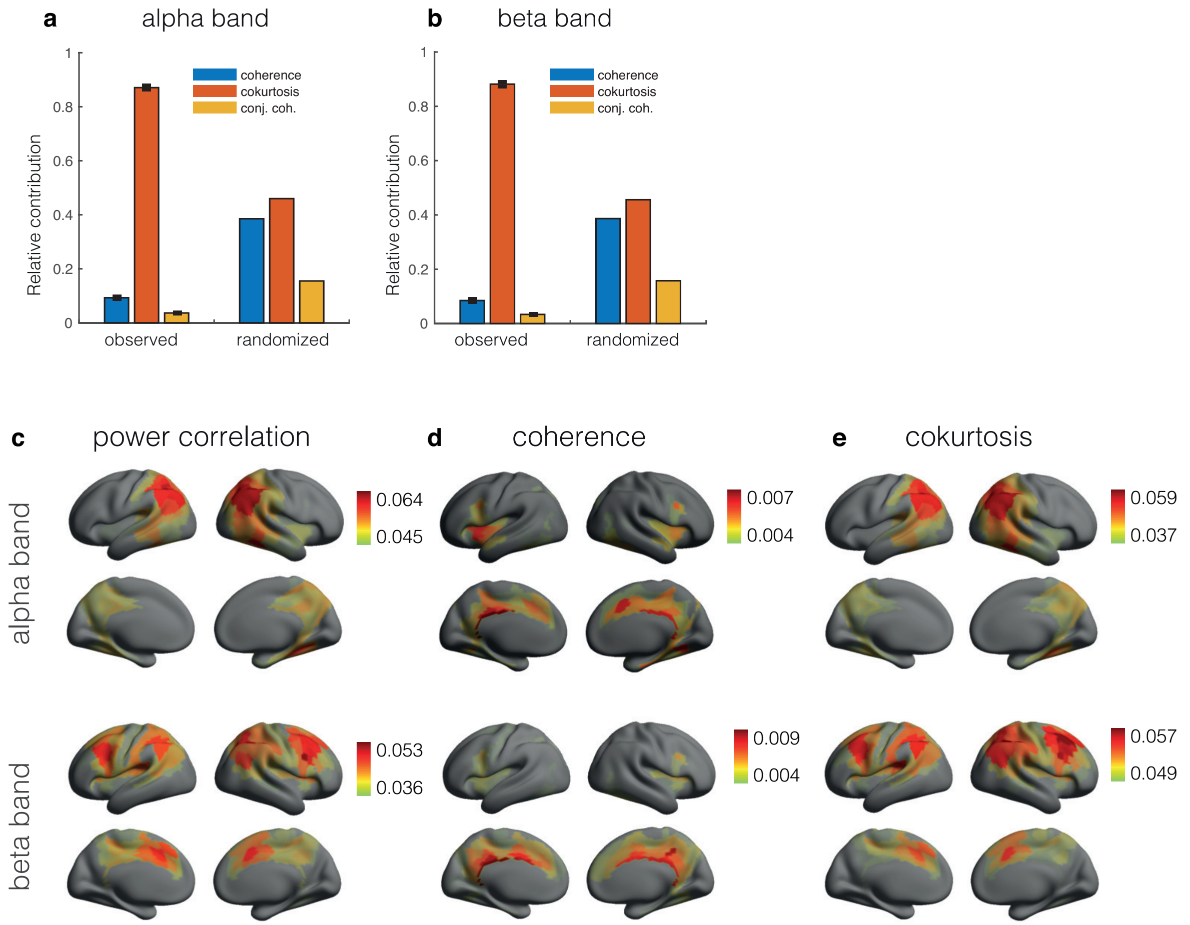

Supplementary Figure 6: Effect of signal orthogonalization (second recording session) (a) Relative contributions of coherence (dark orange bars), cokurtosis (light orange bars), and conjugate coherence (green bars) to cortical power correlations in the alpha frequency band. **(b)** Same format as in (a) but for the beta frequency band. The contributions obtained from a randomized copy of the MEG sensor signals are also shown. The error bars of the observed contributions in panels **(a)** and **(b)** correspond to standard errors and were obtained by bootstrapping over subjects. Error bars for the randomized contributions are not displayed since their standard errors were negligible (less than 0.001). **(c)** Color-coded cortical maps of the region-averaged power correlation network in the alpha (top) and beta (bottom) frequency bands. **(d)** Color-coded cortical maps of the region-averaged coherence network in the alpha (top) and beta (bottom) frequency bands. **(e)** Color-coded cortical maps of the region-averaged cokurtosis network in the alpha (top) and beta (bottom) frequency bands. **Reproducibility:** The correlations between the cortical maps obtained from the first and second recording sessions were 0.96 (power correlation), 0.94 (coherence), and 0.96 (cokurtosis), which shows that the maps are highly reproducible across recording sessions.
